# SILAC labeling coupled to shotgun proteomics analysis of membrane proteins of liver stem/hepatocyte allows to candidate the inhibition of TGF-beta pathway as causal to differentiation

**DOI:** 10.1186/1477-5956-12-15

**Published:** 2014-03-15

**Authors:** Claudia Montaldo, Carmine Mancone, Alice Conigliaro, Angela Maria Cozzolino, Valeria de Nonno, Marco Tripodi

**Affiliations:** 1 National Institute for Infectious Diseases L. Spallanzani, IRCCS, via Portuense 292, 00149 Rome, Italy; 2 Istituto Pasteur-Fondazione Cenci Bolognetti, Department of Cellular Biotechnologies and Haematology, Sapienza University of Rome, Via Regina Elena 324, 00161 Rome, Italy

**Keywords:** Stem cell, Hepatocyte, SILAC, Proteomics

## Abstract

**Background:**

Despite extensive research on hepatic cells precursors and their differentiated states, much remains to be learned about the mechanism underlying the self-renewal and differentiation.

**Results:**

We apply the SILAC (stable isotope labeling by amino acids in cell culture) approach to quantitatively compare the membrane proteome of the resident liver stem cells (RLSCs) and their progeny spontaneously differentiated into epithelial/hepatocyte (RLSCdH). By means of nanoLC-MALDI-TOF/TOF approach, we identified and quantified 248 membrane proteins and 57 of them were found modulated during hepatocyte differentiation. Functional clustering of differentially expressed proteins by Ingenuity Pathway Analysis revealed that the most of membrane proteins found to be modulated are involved in cell-to-cell signaling/interaction pathways. Moreover, the upstream prediction analysis of proteins involved in cell-to-cell signaling and interaction unveiled that the activation of the mesenchymal to epithelial transition (MET), by the repression of TGFB1/Slug signaling, may be causal to hepatocyte differentiation.

**Conclusions:**

Taken together, this study increases the understanding of the underlying mechanisms modulating the complex biological processes of hepatic stem cell proliferation and differentiation.

## Background

Stem/precursor cells are able to differentiate into almost every cell type and are therefore promising tools for the development of new therapeutic approaches in regenerative medicine [1]. Much attention has focused on understanding stem cell biology and the regulation of differentiation to help realize these clinical aspirations [2,3]. However, the key biochemical networks controlling the stem cell biology remain poorly described or undefined, contributing to a lack of knowledge of the principle events regulating differentiation processes.

Quantitative proteomics allows to gather unbiased wide range evidences instrumental to gain deeper insight into the regulatory networks controlling cellular biology [4,5]. This is now possible by integrating proteomic data sets with literature databases [6]. With respect to stem/precursor cells, characterization of molecular pathways causal for self-renewal and multi-step differentiation is desirable. Recent successful studies have allowed for addressing these issues in precursors of many histotypes including self-renewing and differentiating embryonic stem cells [7], neural stem cell differentiation toward astroglial [8] and primary cultured hepatocytes at different stages of development [9].

In this work, we compared the proteome profile of a stem/precursor cell directly with its differentiated progeny: resident liver stem cells (RLSCs) were compared to their progeny spontaneously differentiated into epithelial/hepatocyte (RLSCdH) [10].

RLSCs, previously isolated from murine liver explants, were shown to be non-tumorigenic multi-potent Sca1+ CD34-, CD45-, albumin- stem cell lines capable of i) self-renewal ii) spontaneous differentiation into hepatocytes and cholangiocytes iii), and inducible to differentiating into mesenchymal and neuro-ectodermal cell lineages such as osteoblasts/osteocytes, chondrocytes, astrocytes and neural cells when cultured in appropriate conditions [10].

Moreover, at later stages of differentiation, RLSCdH were shown to switch from hepatocytes bearing a periportal phenotype to perivenular hepatocytes in dependence to Wnt pathway activation [11]. More recently, in orthotropic transplants, RLSCs have also been shown to generate epithelial and mesenchymal liver-specific derivatives (i.e. hepatocytes and hepatic stellate cells) properly integrated in the liver architecture [12].

Here, we aimed to identify RLSC functional elements underlying either self-renewal or differentiation toward hepatocytes. We therefore compared the membrane protein profile of self-renewing RLSCs with those of RLSCdH by using SILAC-based quantitative proteomics. The expression of 57 membrane-associated proteins was found differently regulated. We then systematically studied the differentially expressed membrane proteins. The data highlight the interaction networks and metabolic pathways involved in hepatic stem cell proliferation and differentiation.

## Results

### Identification and quantification of membrane proteins of RLSCs and RLSCdH

Cell growth of stem cells is often a major obstacle for metabolically labeled-based quantitative proteomic analysis. Since RLSCs display long term self-renewing capability, they provide a suitable cellular model to address by SILAC-based proteomics studies of molecular mechanisms controlling stem cell maintenance and differentiation.

**Figure 1:**
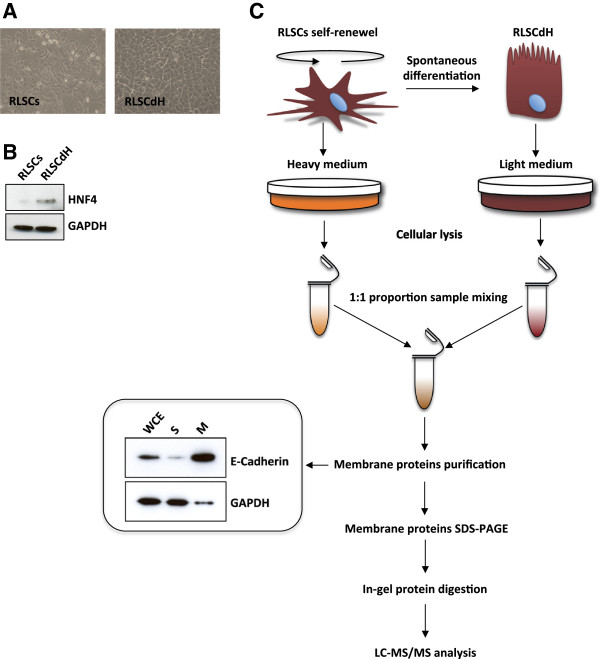
**Schematic representation of SILAC-based proteomic approach on membrane proteins of differentiating RLSCs. (A)** Phase contrast of RLSC and RLSCdH, magnification 20X. **(B)** Western blot analysis in total cell extract for the indicated proteins. For each fraction, 10 μg of protein sample were loaded; Hepatocyte nuclear factor 4 (HNF4) was used to assess cellular differentiation of RLSCs into RLSCdH; and GAPDH was used as proteins loading control. One representative experiment out of three is shown. **(C)** Whole cell extract (WCE), soluble (S) and membrane (M) fractions of mixed RLSC and DH protein samples were analyzed by western blotting. E-cadherin and GAPDH were used respectively as membrane and soluble markers to assay the membrane protein purification.

A self-renewing RLSCs population was long-term metabolically labeled with ^13^C_6_^15^N_4_-arginine and ^13^C_6_-lysine (Heavy) while its differentiated cellular counterpart RLSCdH was grown in light medium (Figures 1A and 1B). Whole cell extracts were then isolated separately and equal amounts of protein from each cell line were mixed and subjected to native membrane purifications by means of Membrane Protein Extraction Kit (M-PEK, Calbiochem). As shown in Figure 1C, a highly enriched membrane protein fraction was obtained. This is mainly due to the membrane isolation kit used in the experiment. In fact, in contrast to the two-phase partitioning technique, where detergents are used to separate membrane proteins based primarily on their intrinsic hydrophobicity, the kit’s scalable differential extraction procedure selectively extracts integral membrane and membrane-associated proteins based on their actual association with cellular membrane.

Membrane proteins were then separated by SDS-PAGE and the gel lane was cut into 16 sections ranging from 15 to 250 kDa. Proteins in each gel section were then digested and submitted to nanoLC-MALDI-TOF/TOF analysis. Proteins were considered identified when at least two peptides were found fragmented by MS/MS, and were considered differentially expressed when the SILAC ratio was Heavy/Light or Light/Heavy ≥1.5. These criteria allowed for listing a total of 57 proteins (~23% of the total proteins identified) (Additional file 1); 31 were found overexpressed in RLSCs and 26 were found overexpressed in RLSCdH. Interestingly, by comparing the membrane proteomic data set with that obtained by means of SILAC approach on the whole cell extracts, we found that differences in membrane-associated protein expression between RLSCs and RLSCdH was due not only by changes in total cellular expression but also as the result of a different cellular localization (Additional file 2).

### Validation of the expression of selected cell surface proteins

**Figure 2:**
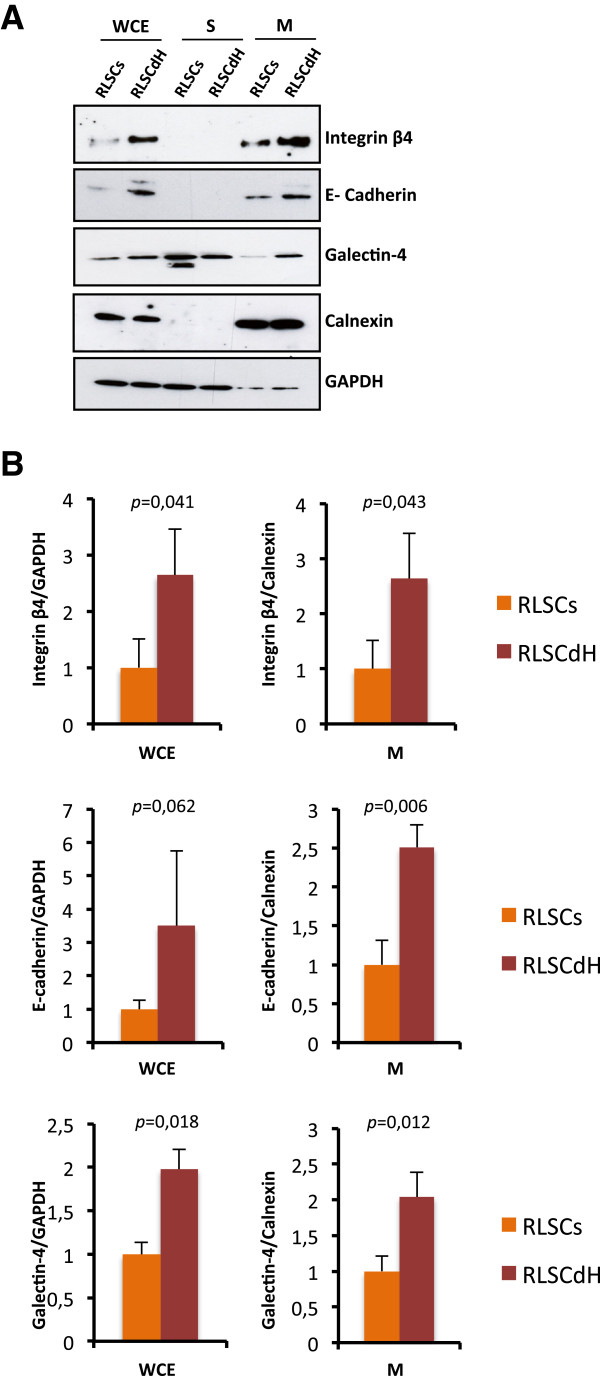
**Validation of the expression of selected cell surface proteins in RLSCs and DH. (A)** Western blot analysis in total cell extract (WCE), soluble (S) and membrane (M) fractions for the indicated proteins. For each fraction, 10 μg of protein sample were loaded; GAPDH and Calnexin expressions were used respectively as total and membrane proteins loading control. One representative experiment out of three is shown. **(B)** Bands were analyzed by densitometry using Quality-One software (Bio-Rad laboratories, Richmond, CA). The Y axis shows the relative intensity respect to GAPDH (WCE) and calnexin (M). All data were from at least three independent experiments and shown as mean ± SD.

Among the 57 proteins quantified, proteins associated to plasma membrane and with a measured fold of variation higher than 2.0 were considered for validation (Table 1). As shown in Figure 2, by means of western blotting analysis we found that the membrane expression levels of e-cadherin, integrin beta-4 and galectin-4 were similar to those measured by quantitative proteomics.

**Table 1:** Differentially expressed cell surface proteins during hepatocyte differentiation of RLSC

**Accession number**^**a**^	**Protein name**	**Fold change DH/RLSC**	**Function**
P09803	E-cadherin	3.6	Cell adhesion
A2A863	Integrin beta-4	2.6	Cell adhesion
Q8K419	Galectin-4	2.0	Cell adhesion

^a^According to Uniprot.

### Bioinformatic analysis of the differentially expressed membrane proteins

**Figure 3:**
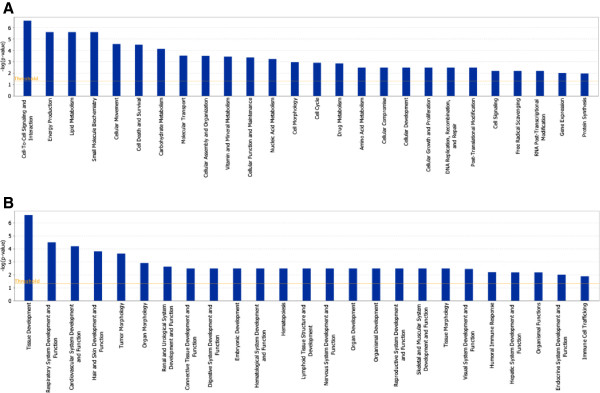
**Biological function analysis.** Molecular and cellular function analysis **(A)** and physiological system development and function analysis **(B)** of differential expression of membrane proteins under hepatocytic differentiation of RLSCs. The significant threshold (*p* value < 0,05) lines are shown.

In order to unveil the molecular functions underlying RLSC differentiation, identified proteins were functionally grouped according to the Ingenuity Pathways Analysis literature database. All the differentially expressed proteins were therefore uploaded to the IPA server. Firstly, we analyzed our proteomic data set in the frame of molecular and cellular functions. Cell-to-cell signaling/interaction, energy production, lipid metabolism, small molecule biochemistry, cellular movement, and cell death and survival were the most significant listed functions, as determined by the *p* value (Figure 3A). Interestingly, proteins involved in the cell-to-cell signaling/interaction processes were listed as a top-listed function (with a significant *p* value <0.000001) while the tissue development was indicated as top-listed (*p* < 0.000001) for physiological system development and function, (Figure 3B).

**Figure 4:**
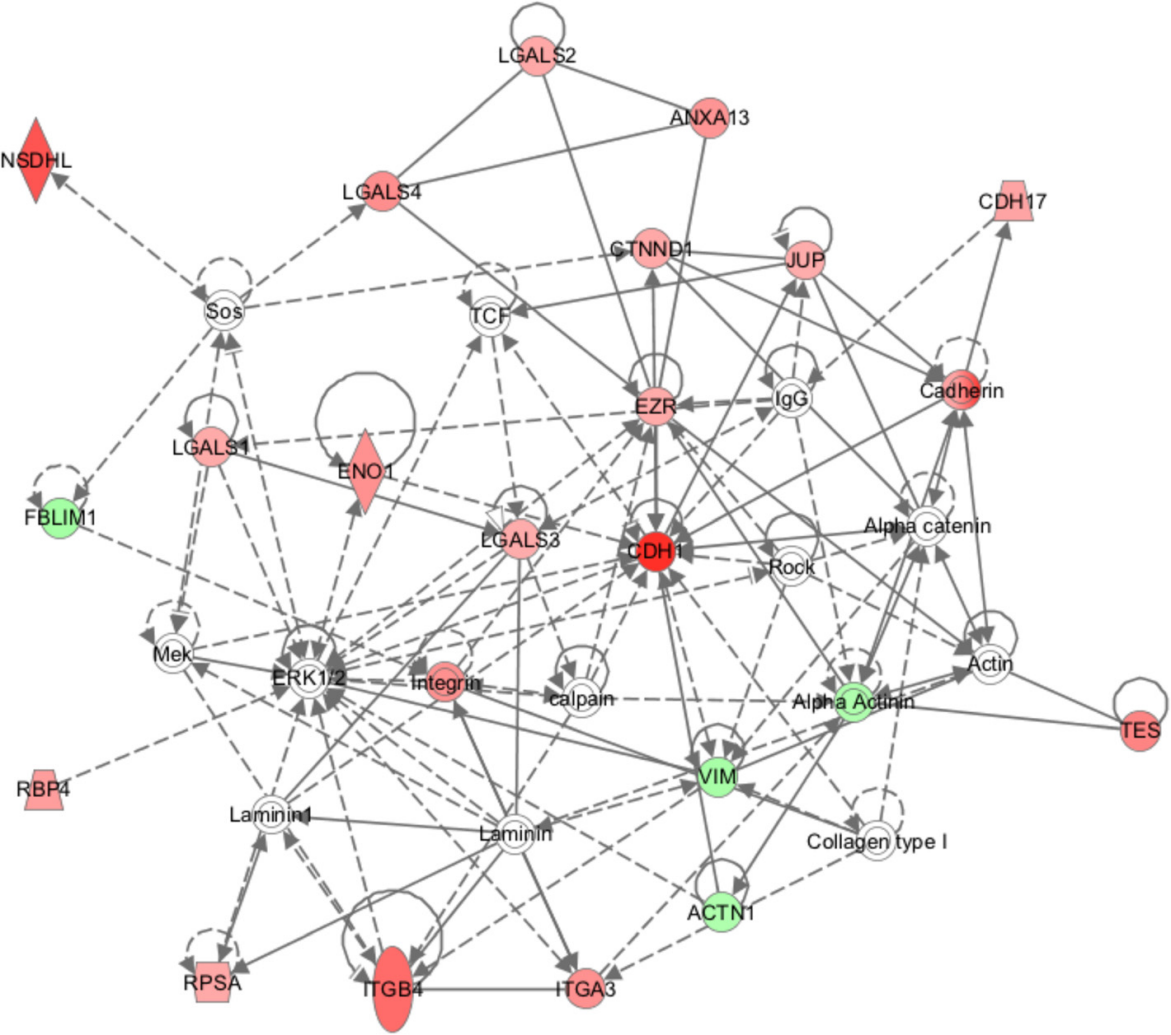
**Molecular pathway analysis of regulated membrane protein changes in RLSCs as they undergo hepatocytic differentiation.** The shown network (Network 1) reveals protein interactions involved in cell-to-cell signaling and interaction, tissue development and cellular movement processes. Red represents an increase in protein expression, whereas green represents a decrease in expression level; the color intensity represents the degree of abundance change. A solid line indicates a direct interaction, and a dashed line indicates an indirect interaction.

Secondly, pathway analysis was also used to analyze different functional networks. Four networks related to previously described functions were generated. Particularly, 23 membrane proteins involved in cell-to-cell signaling and interaction, tissue development and cellular movement were grouped as top 1 network, (IPA score 46) (Figure 4), thus suggesting that these molecular and cellular functions orchestrate the differentiation of RLSCs.

**Figure 5:**
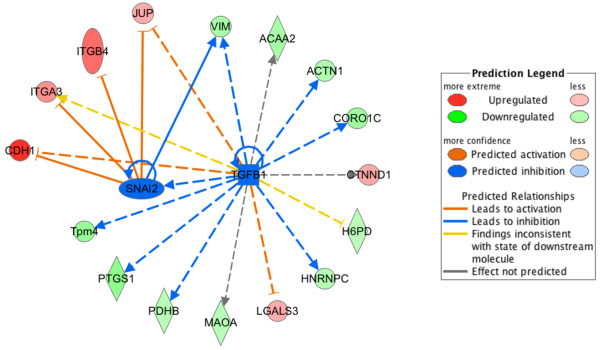
**Upstream regulator analysis of membrane protein changes in differently regulated proteins involved in cell-to-cell signaling and interaction.** Transforming growth factor beta 1 and the transcriptional factor Slug were identified with significant predicted activation score respectively of −2,20 and −2,22. Symbols are indicated in the predicted legend.

Notably, we performed a further IPA analysis in cell-to-cell signaling and interaction to predict upstream molecules, including growth factors and transcription regulators, which may be causal to the observed protein expression changes (Additional file 3). This analysis suggested that the expression of 16 of these proteins could be promoted by the inhibition of transforming growth factor beta 1 (TGFB1) and the transcriptional repressor Slug (SNAI2) (Figure 5). In fact, we observed up-regulation of E-cadherin (CDH1), Integrin alpha-3 (ITGA3), Integrin beta-4 (ITGB4), Junction plakoglobin (JUP) and down-regulation of vimentin (VIM); all these modulations are coherently related to mechanisms underlying the activation of mesenchymal to epithelial transition (MET) and in turn this transition requires the repression of TGFB1/Slug signaling.

## Discussion

Stem/precursor cell differentiation into epithelial cells and persistent maintenance of epithelial phenotype are processes tightly regulated by membrane protein signaling pathways [8,13]. Here, by means of SILAC-based proteomic approach, we compared the membrane proteome of self-renewing RLSCs and their epithelial progeny RLSCdH. This allowed us to quantify a differential protein expression as well as, following computational biology approaches, predict signaling events causal to hepatocyte differentiation.

Among the proteins found to be overexpressed in the RLSCdH, we identify and quantify high levels of three proteins involved in cell adhesion: E-cadherin, integrin beta-4 and galectin-4. Since these proteins have been well-characterized as markers of epithelial polarity [14-16], our findings are in line with our previous report describing the epithelial features of RLSCdH [11].

Our proteomic data set has also been analyzed to unveil potential changes in molecular functions. Through IPA analysis, we found that hepatocyte differentiation leads to extensive changes in cell-to-cell signaling and interaction protein expression. These changes, when functionally correlated, revealed how cell surface-driven signaling may drive the differentiation of liver precursor cells. Interestingly, proteins involved in cell adhesion or motility (Figure 4), including E-cadherin, integrin beta-4, junction plakoglobin and vimentin, have been reported to be directly related to the MET [17-20]. This is consistent with previous reports stating that the activation of MET signaling regulates the balance between embryonic stem cell self-renewal and differentiation [21]. The activation of MET and the reverse process of epithelial to mesenchymal transition (EMT) is regulated by the balance of different master genes. In particular, the liver-enriched transcription factors (LEFTs) like HNF4 are known to be master genes of epithelial/hepatocyte differentiation while the expression of members of the transcriptional repressors belonging to the Snail family regulates the induction of the EMT program and acquisition of stemness traits [22,23]. Recently, it has emerged that members of these families are capable of directly repressing each other and regulating target genes involved in stemness and hepatocyte differentiation in an opposite manner [24]. Here, by IPA up-stream analysis, we unveil that the inhibition of TGFβ, leading to the inhibition of SLUG transcriptional repressors belonging to the Snail family [25], could be responsible for MET activation in hepatocyte differentiation.

## Conclusions

Quantitative SILAC measurement of membrane-associated proteins during the hepatocyte differentiation of RLSCs allowed to reveal signaling pathways activated during the hepatocyte differentiation process. In particular, these findings suggest a direct link between the mesenchymal to epithelial transition and the gain of epithelial properties during stem cell differentiation. This helps to gain insight into potential regulatory networks involved in both the maintenance of the hepatic stem cells multi-potent state and the processes of specific cell lineage differentiation.

## Methods

Information on reagents and proteomic procedures are described in Additional file 4.

### Cell culture and SILAC labeling

All cells were maintained on collagen coating dish at 37°C in 5% CO_2_. RLSC and RLSCdH cell lines were respectively grown in DMEM SILAC “heavy” (^13^C_6_^15^N_4_-arginine and ^13^C_6_-lysine) and RPMI SILAC “light” (^12^C_6_^14^N_4_-arginine and ^12^C_6_-lysine), supplemented with 10% fetal bovine serum (FBS) with recombinant human insulin (10 mg/ml), EGF (50 ng/ml), IGF II (30 ng/ml), for 8 passages before the experiment. This period lasted about 3 weeks, where the SILAC “heavy” cells’ labeling was complete.

Then, all cells were lysed and membrane proteins were isolated following the Membrane Protein Extraction Kit (M-PEK) protocol. Samples were analyzed by Bradford assay to determine the protein concentration, then dissolved in SDS-loading buffer and were analyzed by Western blotting. Equal amounts (155 μg) of membrane proteins from RLSC and RLSCdH cell lines were mixed and 100 μg of this were subsequently analyzed for proteomic analysis as described below. SILAC labeling and proteomic analysis were performed twice.

### Protein digestion and peptide purification

100 μg of membrane proteins obtained from the SILAC experiment were separated on 4 − 12% gradient gels (Invitrogen), stained by Simply Blue Safe Stain staining and visualized. Sixteen sections of the gel lane were cut. Protein-containing gel pieces were washed with 100 μL of 0.1 M ammonium bicarbonate (5 min at RT). Then, 100 μL of 100% acetonitrile (ACN) was added to each tube and incubated for 5 min at RT. The liquid was discarded, the washing step repeated once more, and the gel plugs were shrunk by adding ACN. The dried gel pieces were reconstituted with 100 μL of 10 mM DTT/0.1 M ammonium bicarbonate and incubated for 40 min at 56°C for cysteine reduction. The excess liquid was then discarded and cysteines were alkylated with 100 μL of 55 mM IAA/0.1 M ammonium bicarbonate (20 min at RT, in the dark). The liquid was discarded, the washing step was repeated once more, and the gel plugs were shrunk by adding ACN. The dried gel pieces were reconstituted with 12.5 ng/μL trypsin in 50 mM ammonium bicarbonate and digested overnight at 37°C. The supernatant from the digestion was saved in a fresh tube and 100 μL of 1% TFA/30% ACN were added on the gel pieces for an additional extraction of peptides. The extracted solution and digested mixture were then combined and vacuum centrifuged for organic component evaporation. Peptides were resuspended with 40 μL of 2.5% ACN/0.1% TFA, desalted and filtered through a C18 microcolumn ZipTip, and eluted from the C18 bed using 10 μL of 80% ACN/0.1% TFA. The organic component was once again removed by evaporation in a vacuum centrifuge and peptides were resuspended in a suitable nanoLC injection volume (typically 3–10 μL) of 2.5% ACN/0.1% TFA.

### Data analysis

Differentially expressed proteins were analyzed using Ingenuity Pathway Analysis (IPA, Ingenuity Systems; see http://www.ingenuity.com). The over-represented biological processes, molecular functions, and canonical pathways were generated based on information contained in the Ingenuity Pathways Knowledge Base. Right-tailed Fisher’s exact test was used to calculate a p-value determining the probability that each biological function and/or disease involved in that proteome profile alteration is due to chance alone.

### Western blotting assay

Proteins were separated on Bis-Tris 4 − 12% gradient polyacrylamide gels (Invitrogen) and transferred on nitrocellulose membranes. Membranes were then blocked with 0.5% Tween-PBS containing 5% nonfat dried milk and incubated overnight with the primary antibodies followed by incubation with HRP-conjugated species-specific secondary antibodies and enhanced chemiluminescence reaction using ECL Plus Western Blotting Detection System.

## Competing interest

The authors declare that they have no competing interests.

## Authors’ contributions

ClM and CM designed the study, carried out the cell cultures, performed and analyzed the proteomic analysis. MT, AC, VdN and AMC participated in the study design and analyzed the data. CM and MT conceived the study and drafted the manuscript. All authors have read and approved the final manuscript.

## Supplementary Material

Additional file 1 List of protein identification and SILAC-based quantitation on membrane-associated proteins. Click here for file

Additional file 2: **List of protein identification and SILAC-based quantitative on WCE-associated proteins.** Comparative analysis between SILAC quantitation of membrane and WCE-associated proteins. Click here for file

Additional file 3 List of upstream regulators of membrane protein changes in RLSCs when they undergo hepatocytic differentiation. Click here for file

Additional file 4 Additional Methods. Click here for file
