# Supplementary material for: SILAC labeling coupled to shotgun proteomics analysis of membrane proteins of liver stem/hepatocyte allows to candidate the inhibition of TGF-beta pathway as causal to differentiation
Source: Proteome Sci. 2014 Mar 15;12:15. doi: 10.1186/1477-5956-12-15 (PMC4007997; doi:10.1186/1477-5956-12-15)
Supplement: Additional file 4 — Additional Methods. [file 1477-5956-12-15-S4.docx]

**Supplemental data 4**

**Reagents**

The chemicals, reagents and suppliers that were used are listed below: N,N,N,N-tetrametiletilenediammine (TEMED), Sodium Dodecyl Sulfate (SDS), Tween 20, Albumin Serum Bovine (BSA), Phosphate Buffered Saline (PBS), Dulbecco’s modified eagle medium (DMEM), RPMI-1640 medium, Trypsin-EDTA solution, Fetal Bovine Serum (FBS), L-Glutamine, penicillin/streptomycin, trifluoroacetic acid, Ponceau S solution, α-cyano-4-hydroxycinnamic acid (CHCA), protease inhibitor cocktail (PIC), dl-dithiothreitol (DTT), iodoacetamide (IAA) and ammonium bicarbonate (NH4HCO3) were purchased from Sigma-Aldrich (St. Louis, MO). Recombinant human insulin was purchased from Roche (Mannheim, Germany), recombinant human EGF and IGF-II were purchased from Peprotech (Hamburg, Germany). Bradford Bio-Rad Protein Assay, Page Ruler tm Plus Prestained Protein Ladder Fermentas Life Sciences (Vilnius, Lithuania). NuPAGE gel electrophoresis reagents Simply Blue Safe Stain and Gibco Fetal Bovine Serum were obtained from Life Technologies (Carlsbad, CA). SILAC media DMEM was purchased from Silantes (Munich, Germany). Nytrocellulose membranes (Protran BA85) were obtained from Whatman-Schleicher & Schuel (Dassel, Germany). ECL Plus Western Blotting Detection System was purchased from GE Healthcare (Chalfont St Giles, U.K.). Methanol and acetonitrile (ACN) were purchased from J.T. Baker (Phillipsburg, NJ) and Microcolumn ZipTip (μ-C18) were purchased from Millipore Corporation (Ballerica, USA). Anti-Calnexin, anti-Galectin 4 and anti-Integrin B4 antibodies were purchased from Santa Cruz (Dallas, Texas). Anti-E Cadherin antibody was purchased from BD Bioscences Pharmingen (Europe). Anti-GAPDH antibody and Membrane Protein Extraction Kit (M-PEK) was purchased from Calbiochem Merck (Gibbstown, NJ).

**nanoLC Analysis and Mass Spectrometry Analysis**

An UltiMate 3000 nano-LC system (Dionex, Sunnyvale, CA) equipped with an integrated nanoflow manager and micro-vacuum degasser was used for peptide separation. The peptides were loaded onto a 75 μm I.D. NanoSeries C18 column (Dionex, P/N 160321) for multistep gradient elution (eluent A 0.05% TFA; eluent B 0.04% TFA in 80% ACN) from 5 to 20% eluent B within 10 min, from 20 to 50% eluent B within 45 min and for further 5 min from 50 to 90% eluent B with a constant flow of 0.3 μL/min. After 5 min, the eluted sample fractions were continuously diluted with 0.5 μL/min a-cyano-4-hydroxycinnamic acid (CHCA) and spotted onto a MALDI target using a Probot (LC-Packings/Dionex) with an interval of 20 s resulting in 144 fractions for each gel slice.

MALDI-TOF-MS spectra were acquired using a 4800 Plus MALDI TOF/TOF Analyzer (AB Sciex, Foster City, CA). The spectra were acquired in the positive reflector mode by 20 subspectral accumulations (each consisting of 50 laser shots) in an 800−4000 mass range, focus mass 2100 Da, using a 355 nm Nb:YAG laser with a 20 kV acceleration voltage. Peak labeling was automatically done by 4000 Series Explorer software Version 3.0 (AB Sciex) without any kind of smoothing of peaks or baseline, considering only peaks that exceeded a signal-to-noise ratio of 10 (local noise window 200 m/z ) and a half maximal width of 2.9 bins. Calibration was performed using default calibration originated by five standard spots (ABI4700 Calibration Mixture). Only MS/MS spectra of preselected peaks (out of peak pairs with a mass difference of 6.02, 10.01, 12.04, 16.03, and 20.02 Da) were integrated over 1000 laser shots in the 1 kV positive ion mode with the metastable suppressor turned on. Air at the medium gas pressure setting (1.25×10−6 Torr) was used as the collision gas in the CID off mode. After smoothing and baseline subtractions, spectra were generated automatically by 4000 Series Explorer software. MS and MS/MS spectra were processed by ProteinPilot Software 2.0.1 (AB SCIEX) which acts as an interface between the Oracle database containing raw spectra and a local copy of the MASCOT search engine (Version 2.1, Matrix Science, Ltd).

The Paragon algorithm was used with SILAC (Lys+6, Arg+10) selected as the Sample Type, iodacetamide as cysteine alkylation, with the search option “biological modifications” checked, and trypsin as the selected enzyme. MS/MS protein identification was performed against the UniProtKB/TrEMBL mouse database (48701576 sequence entries; Release 20131211), using a confidence threshold of 95% (Proteinpilot Unused score ≥ 1.31). The monoisotopic precursor ion tolerance was set to 0.12 Da and the MS/MS ion tolerance to 0.3 Da. The minimum required peptide length was set to 6 amino acids; two peptides were required for protein identification. All accepted human peptides had a false discovery rate (FDR) of 0.05 using reversed database searches. For quantitation, the Heavy/Light average ratio for a protein was calculated by ProteinPilot Software with automatic bias correction. Quantitation was based on a two-dimensional centroid of the isotope clusters within each SILAC pair. Ratios of the corresponding isotope forms in the SILAC pair were calculated, and lines fitting these intensity ratios gave the slope as the desired peptide ratio. Only relative Heavy/Light (or Light/Heavy) ratios exceeding factor 2.0 were considered.
